# Vascular Risk Factors and Cognition in Multiple System Atrophy

**DOI:** 10.3389/fnins.2021.749949

**Published:** 2021-10-26

**Authors:** Lingyu Zhang, Yanbing Hou, Bei Cao, Qian-Qian Wei, Ruwei Ou, Kuncheng Liu, Junyu Lin, Tianmi Yang, Yi Xiao, Bi Zhao, HuiFang Shang

**Affiliations:** Laboratory of Neurodegenerative Disorders, Department of Neurology, Rare Diseases Center, National Clinical Research Center for Geriatrics, West China Hospital, Sichuan University, Chengdu, China

**Keywords:** multiple system atrophy, cognition, vascular risk factor, non-motor symptom, neurodegenerative disorder

## Abstract

**Objective:** Vascular risk factors have been reported to be associated with cognitive impairment (CI) in the general population, but their role on CI in multiple system atrophy (MSA) is unclear. This study aimed to explore the relationship between vascular risk factors and CI in patients with MSA.

**Methods:** The clinical data and vascular risk factors were collected. The Montreal Cognitive Assessment tool was used to test the cognitive function of patients with MSA. Binary logistic regression was used to analyze the correlation between vascular risk factors and CI.

**Results:** A total of 658 patients with MSA with a mean disease duration of 2.55 ± 1.47 years were enrolled. In MSA patients, hypertension was recorded in 20.2%, diabetes mellitus in 10.3%, hyperlipidemia in 10.2%, smoking in 41.2%, drinking in 34.8%, and obesity in 9.6%. The prevalence of CI in patients with MSA, MSA with predominant parkinsonism (MSA-P), and MSA with predominant cerebellar ataxia (MSA-C) was 45.0, 45.1, and 44.9%, respectively. In the binary logistic regression model, patients with more than one vascular risk factors were significantly more likely to have CI in MSA (OR = 4.298, 95% CI 1.456–12.691, *P* = 0.008) and MSA-P (OR = 6.952, 95% CI 1.390–34.774, *P* = 0.018), after adjusting for age, sex, educational years, disease duration, and total Unified multiple system atrophy rating scale scores.

**Conclusion:** Multiple vascular risk factors had a cumulative impact on CI in MSA. Therefore, the comprehensive management of vascular risk factors in MSA should not be neglected.

## Introduction

Multiple system atrophy (MSA) is a sporadic neurodegenerative disease clinically characterized by the combination of Parkinsonian, cerebellar, autonomic, or pyramidal signs and symptoms (Stefanova et al., 2009). The pathological hallmark of MSA is the presence of oligodendrocytic glial cytoplasmic inclusions consisting of α-synuclein. Patients with MSA only have a mean survival period of about 7–9 years after initial clinical presentation (Stefanova et al., 2009). However, the etiology of MSA is still unclear. Currently, symptomatic treatment is the only therapeutic option since disease-modifying therapy is not available.

Urinary failure, erectile dysfunction, orthostatic hypotension, sleep disorders, mood disorders, and cognitive dysfunction are common non-motor symptoms in MSA (Schrag et al., 2010; Cao et al., 2015b; Zhang et al., 2017). Cognitive dysfunction had been underestimated previously, but an increasing number of studies have reported that cognitive impairment (CI) can present as a single-domain deficit or as a wide spectrum of domains (Stankovic et al., 2014; Cao et al., 2015b; Lee et al., 2015). Specifically, frontal executive dysfunction is the most commonly affected domain, followed by the visuospatial, memory, and attention domains (Stankovic et al., 2014; Cao et al., 2015b). In addition, CI has been reported in autopsy-confirmed MSA patients (Wenning et al., 1997). A recent study reported that MSA patients with CI had a greater burden of neuronal cytoplasmic inclusions in the limbic regions (the dentate gyrus) (Koga et al., 2017).

Previous studies have found that vascular risk factors, such as smoking and alcohol drinking, were associated with CI at late life in the general population (Wu et al., 2018). Hypertension, hypercholesterolemia, and diabetes have also been reported to be associated with dementia in middle-aged people (Kivipelto et al., 2006). Vascular risk factors were associated with CI in patients with Parkinson’s disease (PD) (Malek et al., 2016; Pilotto et al., 2016). Smoking was probably a protective factor in MSA (Vanacore et al., 2000, 2001; Vanacore, 2005), and increasing alcohol consumption may decrease the risk of MSA (Vidal et al., 2008). Levels of serum cholesterol have been reported to be insignificantly correlated with disease duration or severity, but low levels of total cholesterol and high-density lipoprotein may be associated with an increased risk of MSA (Lee et al., 2009). Our previous studies have shown that low levels of uric acid and severe motor symptoms were related to CI in patients with MSA (Cao et al., 2015a, b). However, the relationship between vascular risk factors and cognition has never been specifically studied in MSA. As such, this study aimed to provide a detailed prevalence of the vascular risk factors in MSA and evaluate the correlation between these vascular risk factors and CI in MSA.

## Materials and Methods

### Patients

Consecutive patients with a clinical diagnosis of MSA and evaluated at the Department of Neurology, West China Hospital of Sichuan University between August 2013 and Jun 2021 were included in the current study. According to the second consensus criteria, the diagnosis of MSA was divided into three groups (Gilman et al., 2008). Definite MSA requires the neuropathologic demonstration of CNS α-synuclein–positive glial cytoplasmic inclusions with neurodegenerative changes in the striatonigral or olivopontocerebellar structures. Probable MSA requires a sporadic, progressive adult-onset disorder, including rigorously defined autonomic failure and parkinsonism or cerebellar ataxia that is poorly responsive to levodopa. Lastly, possible MSA requires a sporadic, progressive adult-onset disease, including parkinsonism or cerebellar ataxia, and at least one feature suggesting autonomic dysfunction plus one other feature that may be a clinical or a neuroimaging abnormality. Only patients diagnosed with probable MSA were included in the final analysis. Patients with predominantly parkinsonian features were designated as MSA-P, and patients predominantly presenting with cerebellar ataxia were designated as MSA-C. All patients included underwent magnetic resonance imaging (in our or other external hospitals) to exclude prominent cortical or subcortical infarcts, iron accumulation, or other atypical parkinsonian disorders. In order to exclude the common forms of spinocerebellar ataxia (SCA), patients were screened for SCA genes, including SCA1, 2, 3, 6, and 7.

The clinical data of age, sex, height, weight, educational years, and disease duration were collected by professional neurologists via face-to-face interviews. Disease onset referred to the initial presentation of any motor problems (whether parkinsonism or cerebellar) or autonomic features, except male erectile dysfunction (Gilman et al., 2008). Disease duration referred to the time from the disease onset date to the evaluation date. The Unified multiple system atrophy rating scale (UMSARS) was used to evaluate the disease severity (Wenning et al., 2004). Orthostatic hypotension (OH) was defined as a reduction in the systolic blood pressure (BP) by at least 30 mmHg and/or diastolic BP by at least 15 mmHg 3 min after standing up from a previous recumbent position for 10 min. A comprehensive and standardized cognitive battery (Montreal cognitive assessment) was applied to assess the global cognitive functions. The optimal cutoff scores for cognitive impairment screening were 19 for individuals with no more than 6 years of education, 22 for individuals with 7–12 years of education, and 24 for individuals with more than 12 years of education (Chen et al., 2016).

### Vascular Risk Factors Evaluation

Vascular risk factors were evaluated during the clinical assessment. Hypertension was defined as a systolic BP ≥ 140 mm Hg, diastolic BP ≥ 90 mm Hg, self-reported use of antihypertensive medications, or lifetime diagnosis of hypertension. Nearly half of the patients completed the blood tests in our hospital. Diabetes mellitus was defined as fasting plasma glucose ≥ 126 mg/dL (7.0 mmol/L) for patients completed the blood tests in our hospital, reported use of hypoglycemic agents, or any self-reported history of diabetes. Hyperlipidemia was defined as total cholesterol ≥ 6.2 mmol/L or triglyceride ≥ 2.3 mmol/L for patients completed the blood tests in our hospital, use of lipid-lowering medications, or lifetime diagnosis of hyperlipidemia. Personal history of smoking behavior was indicated by pack/years to quantify the packs smoked per day multiplied by years as a smoker, with the factor threshold set to 15 (Heinzel et al., 2014; Pilotto et al., 2016). Drinking was defined as an average alcoholic drink ≥ 50 mL at least once per week lasting more than half a year. The body mass index (BMI) was calculated as body weight (kg) divided by heights squared (m^2^). Following the Chinese criteria for overweight/obesity, the patients were classified as normal (BMI 18.5–23.9 kg/m^2^), overweight (24–27.99 kg/m^2^), or obese (≥ 28.0 kg/m^2^).

This study was approved by the Ethics Committee of West China Hospital of Sichuan University. Informed consent was obtained from all participants.

### Statistical Analysis

All continuous data are presented as the mean ± standard deviation, while all categorical variables are presented as numbers or percentages. The clinical characteristics and vascular risk factors prevalence of patients with and without CI were compared using the Student’s *t*-test and the χ^2^-test for continuous and dichotomous variables, respectively. A binary logistic regression model was used to explore the potential vascular risk factors related to CI in MSA. The presence or absence of CI was used as the dependent variable. All the vascular risk factors were considered covariables after adjusting for age, sex, subtypes, educational years, disease duration, and total UMSARS scores.

All the data analyses were performed using SPSS 22.0 (IBM, Chicago, IL). A *p*-value < 0.05 was considered statistically significant.

## Results

The demographic and clinical features of patients with MSA are presented in Table 1. Among the 658 patients included in the analysis, the following were observed: mean age of 60.13 ± 8.74 years, mean age at onset of 57.51 ± 8.60 years, and a mean disease duration of 2.55 ± 1.47 years. Furthermore, 57.1% were male (Table 1). In terms of the vascular risk factors, 20.2% of patients had hypertension, 10.3% had diabetes mellitus, 10.2% had hyperlipidemia, 41.2% were cigarette smokers, 34.8% were alcohol drinks, and 9.6% were obese. A total of 435 (66.1%) patients with MSA had at least one vascular risk factor, while 269 (40.95) patients had more than one.

**TABLE 1:** Demographic and clinical features of the patients with MSA.

Variables	MSA
Total	658
Diagnosis (MSA-P, %)	304 (46.2%)
Sex (male, %)	376 (57.1%)
Age	60.13 ± 8.74
Age of onset	57.51 ± 8.60
Educational years	9.63 ± 3.74
BMI	23.63 ± 3.30
Disease duration	2.55 ± 1.47
MoCA score	22.06 ± 4.77
UMSARS-I	16.35 ± 6.48
UMSARS-II	18.11 ± 6.84
UMSARS-IV	2.12 ± 0.98
Total UMSARS scores	34.45 ± 12.47
OH (%)	232 (35.3%)
Hypertension	133 (20.2%)
Diabetes mellitus	68 (10.3%)
Hyperlipidemia	67 (10.2%)
Cigarette smoking	271 (41.2%)
Drinking	229 (34.8%)
Obesity	63 (9.6%)
**Number of vascular risk factors**	
0	223 (33.9%)
1	166 (25.2%)
≥2	269 (40.9%)

*MSA, multiple system atrophy; MSA-P, multiple system atrophy with predominately parkinsonism; CI, cognitive impairment; BMI, body mass index; MoCA, Montreal cognitive assessment; UMSARS, unified multiple system atrophy rating scale; OH, orthostatic hypotension.*

The comparisons of the demographic and clinical features between patients with and without CI in MSA, MSA-P, and MSA-C are shown in Table 2. The prevalence of CI in patients with MSA, MSA-P, and MSA-C was 45.0, 45.1, and 44.9%, respectively. In the MSA, MSA-P, and MSA-C groups, the patients with CI were older; had late age at onset; higher UMSARS-I, UMSARS-II, UMSARS-IV, and total UMSARS scores than the patients without (*P* < 0.05). The proportion of hypertension, diabetes mellitus, hyperlipidemia, cigarette smoking, drinking, and obesity were not significantly different between the patients with and without CI (*P* > 0.05). Patients with CI had a greater number of vascular risk factors than those without, although this was not significantly different.

**TABLE 2:** The comparison of the demographic and clinical features between patients with and without CI in the MSA, MSA-P, and MSA-C groups.

	MSA			MSA-P			MSA-C		
Variables	MSA without CI	MSA with CI	*P*-value	MSA without CI	MSA with CI	*P*-value	MSA without CI	MSA with CI	*P*-value
Total	362 (55.0%)	296 (45.0%)	–	167 (54.9%)	137 (45.1%)	–	195 (55.1%)	159 (44.9%)	–
Diagnosis (MSA-P, %)	167 (46.1%)	137 (46.3%)	0.969	–	–	–	–	–	–
Sex (male, %)	203 (56.1%)	173 (58.4%)	0.541	89 (53.3%)	77 (56.2%)	0.612	114 (58.5%)	96 (60.4%)	0.715
Age	58.67 ± 8.69	61.91 ± 8.47	<0.001*	60.09 ± 9.06	63.48 ± 8.69	0.001*	57.46 ± 8.19	60.56 ± 8.07	<0.001*
Age of onset	56.12 ± 8.62	59.20 ± 8.28	<0.001*	57.31 ± 9.11	60.62 ± 8.62	0.001*	55.11 ± 8.07	57.98 ± 7.80	0.001*
Educational years	9.93 ± 3.74	9.26 ± 3.72	0.024*	9.77 ± 3.76	9.23 ± 3.75	0.215	10.06 ± 3.72	9.29 ± 3.71	0.054
BMI	23.73 ± 3.24	23.51 ± 3.38	0.413	23.94 ± 3.47	23.35 ± 3.66	0.149	23.54 ± 3.02	23.66 ± 3.13	0.724
Disease duration	2.53 ± 1.43	2.57 ± 1.52	0.713	2.71 ± 1.53	2.80 ± 1.69	0.610	2.37 ± 1.33	2.37 ± 1.34	0.983
MoCA score	25.33 ± 2.48	18.05 ± 3.72	<0.001*	25.39 ± 2.48	18.20 ± 3.74	<0.001*	25.28 ± 2.48	17.91 ± 3.72	<0.001*
UMSARS-I	15.23 ± 5.86	17.71 ± 6.92	<0.001*	15.25 ± 6.40	17.97 ± 6.55	<0.001*	15.22 ± 5.38	17.48 ± 7.24	0.001*
UMSARS-II	16.58 ± 6.04	19.98 ± 7.29	<0.001*	17.55 ± 6.53	20.79 ± 7.32	<0.001*	15.75 ± 5.47	19.28 ± 7.22	<0.001*
UMSARS-IV	1.93 ± 0.84	2.36 ± 1.09	<0.001*	2.02 ± 0.92	2.34 ± 1.06	0.005*	1.85 ± 0.76	2.38 ± 1.11	<0.001*
Total UMSARS scores	31.81 ± 11.11	37.69 ± 13.27	<0.001*	32.79 ± 12.29	38.76 ± 12.85	<0.001*	30.97 ± 9.95	36.77 ± 13.59	<0.001*
OH (%)	127 (35.1%)	105 (35.5%)	0.917	40 (24.0%)	44 (32.1%)	0.113	87 (44.6%)	61 (38.4%)	0.236
Hypertension	65 (18.0%)	68 (23.0%)	0.111	34 (20.4%)	34 (24.8%)	0.353	31 (15.9%)	34 (21.4%)	0.185
Diabetes mellitus	38 (10.5%)	30 (10.1%)	0.879	19 (11.4%)	14 (10.2%)	0.747	19 (9.7%)	16 (10.1%)	0.920
Hyperlipidemia	42 (11.6%)	25 (8.4%)	0.183	11 (6.6%)	10 (7.3%)	0.807	31 (15.9%)	15 (9.4%)	0.072
Cigarette smoking	144 (39.8%)	127 (42.9%)	0.418	61 (36.5%)	52 (38.0%)	0.798	83 (42.6%)	75 (47.2%)	0.386
Drinking	122 (33.7%)	107 (36.1%)	0.512	58 (34.7%)	47 (34.3%)	0.938	64 (32.8%)	60 (37.7%)	0.335
Obesity	36 (9.9%)	27 (9.1%)	0.721	20 (12.0%)	13 (9.5%)	0.488	16 (8.2%)	14 (8.8%)	0.840
**Number of vascular risk factors**									
0	136 (37.6%)	87 (29.4%)	0.085	70 (41.9%)	44 (32.1%)	0.152	66 (33.8%)	43 (27.0%)	0.357
1	85 (23.5%)	81 (27.4%)		34 (20.4%)	38 (27.7%)		51 (26.2%)	43 (27.0%)	
≥2	141 (39.0%)	128 (43.2%)		63 (37.7%)	55 (40.1%)		78 (40.0%)	73 (45.9%)	

*MSA, multiple system atrophy; MSA-P, multiple system atrophy with predominately parkinsonism; MSA-C, multiple system atrophy with predominately cerebellar ataxia; CI, cognitive impairment; BMI, body mass index; MoCA, Montreal cognitive assessment; UMSARS, unified multiple system atrophy rating scale; OH, orthostatic hypotension.*

*Significant difference.

The correlations between vascular risk factors and CI in patients with MSA, MSA-P, and MSA-C in the binary logistic regression model are shown in Table 3. Patients with more than one vascular risk factor were significantly more likely to have CI in MSA (OR = 4.298, 95% CI 1.456–12.691, *P* = 0.008) and MSA-P (OR = 6.952, 95% CI 1.390–34.774, *P* = 0.018), after adjusting for age, sex, educational years, disease duration, and total UMSARS scores. However, there was no significant correlation between the number of vascular risk factors and CI in patients with MSA-C.

**TABLE 3:** The correlation between the vascular risk factors and CI in patients with MSA, MSA-P, and MSA-C in the binary logistic regression model.

Variables	MSA			MSA-P			MSA-C		
	OR	95% CI	*P*-value^a^	OR	95% CI	*P*-value^b^	OR	95% CI	*P*-value^b^
Hypertension	0.778	0.453–1.336	0.363	0.701	0.316–1.555	0.383	0.834	0.393–1.772	0.638
Diabetes mellitus	0.587	0.315–1.092	0.093	0.410	0.152–1.104	0.078	0.700	0.305–1.607	0.401
Hyperlipidemia	0.466	0.244–1.107	0.055	0.883	0.308–2.529	0.816	0.353	0.149–1.106	0.059
Cigarette smoking	0.663	0.351–1.253	0.206	0.514	0.202–1.308	0.162	0.854	0.352–2.075	0.728
Drinking	0.582	0.312–1.085	0.089	0.391	0.154–1.101	0.054	0.662	0.275–1.594	0.358
Obesity	0.774	0.413–1.449	0.423	0.542	0.216–1.363	0.193	0.917	0.377–2.234	0.850
**Number of vascular risk factors**									
0	1 (reference)	1 (reference)	–	1 (reference)	1 (reference)	–	1 (reference)	1 (reference)	–
1	2.183	1.206–3.951	0.010*	2.835	1.182–6.799	0.020*	1.713	0.755–3.884	0.198
≥2	4.298	1.456–12.691	0.008*	6.952	1.390–34.774	0.018*	3.363	0.751–15.070	0.113

*MSA, multiple system atrophy; MSA-P, multiple system atrophy with predominately parkinsonism; MSA-C, multiple system atrophy with predominately cerebellar ataxia; CI, cognitive impairment.*

*^a^Adjusted for age, sex, subtypes, educational years, disease duration, and total UMSARS scores.*

*^b^Adjusted for age, sex, educational years, disease duration, and total UMSARS scores.*

*Significant difference.

## Discussion

It has been reported that vascular risk factors such as hypertension, hypercholesterolemia, and obesity play important roles in the development of dementia in the general population (Kivipelto et al., 2006). However, their effect on the cognition of patients with MSA is still unknown. To the best of our knowledge, the present cross-sectional study was the first study to investigate the influence of vascular risk factors on cognition in a large cohort of patients with MSA who underwent a standardized global cognitive assessment, taking their demographic and clinical confounders into account.

In the current study, we found that CI was present in 45.0% of patients with MSA. Combining the subjective CI symptoms and different cognitive screening tests to assess CI in 102 MSA patients, Koga et al. (2017) showed that the prevalence CI was 32%. Fiorenzato et al. (2017) reported that the prevalence of CI was 30.6% in 72 MSA patients, based on the mini-mental state examination score < 27. The prevalences of CI in the above studies were slightly lower compared to our present finding, possibly due to their small sample sizes and difference in the cognitive assessment scales used. The mean disease duration of our patients with CI was 2.57 years. Previous studies showed that the mean disease duration of MSA patients with CI ranged from 1.25 to 7 years (O’Sullivan et al., 2008; Stankovic et al., 2014; Auzou et al., 2015; Lee et al., 2015; Fiorenzato et al., 2017; Koga et al., 2017). The discrepancy could be due to populational bias or variations in the criteria used to access CI. It is noteworthy that CI can appear in an early stage of MSA.

Vascular risk factors are not uncommon in MSA, given that hypertension was recorded in 20.2%, diabetes mellitus in 10.3%, hyperlipidemia in 10.2%, smoking in 41.2%, drinking in 34.8%, and obesity in 9.6%. The prevalences of hypertension and diabetes mellitus in the general Chinese population (age ≥ 60 years) were estimated to be 60.0 and 20.0%, respectively, which were higher compared to our study population (Xu et al., 2013; Wang et al., 2014). The prevalence of hyperlipidemia in adults aged between 35 and 75 years in the Chengdu area was 23.53%, which was higher than in our patients (10.2%) (Liao et al., 2013). Meanwhile, the prevalence of smoking in Chinese adults (aged between 60 and 69 years) was about 38.0%, lower than our patients (41.2%) (Ding et al., 2016). The prevalence of drinking in the general Chinese population (aged between 55 and 65 years) was lower compared to our study population (30.0 vs. 34.8%) (Li et al., 2018). Lastly, the prevalence of obesity in the general Chinese population (aged between 35 and 72) was 14.0%, higher than in our patients (9.6%) (Zheng et al., 2015).

We found that a single vascular risk factor (e.g., hypertension, diabetes mellitus, hyperlipidemia, etc.) was not associated with CI in patients with MSA, MSA-P, and MSA-C. However, the current study showed the cumulative impact of multiple vascular risk factors on CI in MSA. It has been reported that the presence of more than two vascular risk factors was significantly associated with CI in patients with PD (Malek et al., 2016), which can support our results since MSA and PD belong to α-synucleinopathy. However, the pathophysiological mechanisms of the vascular risk factors associated with CI in patients with MSA remain unclear. Therefore, further mechanism studies are needed to elucidate these.

Hypertension has been reported as an important risk factor for the development of CI and dementia (DeCarli, 2015) due to the possible mechanistic endothelial dysfunction or vascular dysregulation, oxidative stress, and inflammation (Gorelick, 2014). Previous studies also suggested that hyperlipidemia was associated with the risk of mild CI and dementia (Carlsson, 2010; Panza et al., 2011). Kivipelto et al. (2006) found that obesity was one of the risk factors present at midlife, which can predict the future risk of dementia in the general population. Obesity is a risk factor for several metabolic diseases, such as insulin resistance and type 2 diabetes. Previous studies have revealed that insulin resistance may be important in the pathogenesis of Alzheimer’s disease (Kuusisto et al., 1997; Watson and Craft, 2003). Furthermore, obesity may increase microglial activation, which has been observed in MSA through positron emission tomography molecular imaging (Niccolini and Politis, 2016). Furthermore, a recent study found that increased microglial activation and dendritic spine loss may be responsible for obesity-associated cognitive decline (Cope et al., 2018). Therefore, the mechanism of obesity involved in CI in MSA, which needs to be confirmed in further researches. Epidemiological studies have demonstrated that changes in lifestyle, including frequent physical exercise, can prevent and treat not only obesity/metabolic disorders but also improve cognitive function through epigenetic mechanisms (Barros et al., 2019). Therefore, physical exercise has been proposed as a non-pharmacological treatment of CI (Barros et al., 2019).

A long-term follow-up study (up to 27 years) found that frequent alcohol consumption was associated with an increased risk for dementia, compared to infrequent alcohol intake (Langballe et al., 2015). Researches have revealed that even moderate alcohol drinking in older people was associated with gray matter atrophy and reduced total brain volume and frontal and parietal gray matter densities (Mukamal et al., 2001; den Heijer et al., 2004; Paul et al., 2008; Sachdev et al., 2008). Similarly, another study analyzing a 30-year longitudinal data focused on the relationship between alcohol consumption and brain structure and function and found that even a moderate alcohol consumption was associated with hippocampal atrophy and cognitive decline (Topiwala et al., 2017). In the Chinese population, studies have shown that alcohol intake was associated with an increased risk of CI (Zhou et al., 2003; Wu et al., 2018). In addition, concomitant smoking and regular alcohol drinking at midlife had a much stronger impact than the individual factors on the risk of CI in late life in the general population (Wu et al., 2018). Therefore, we recommend reducing alcohol intake and smoking in MSA patients. The comprehensive management of multiple vascular risk factors may protect patients with MSA from CI.

The strength of our study was that it was the first study to focus on the prevalence of vascular risk factors and the relationship between vascular risk factors and cognition in a large sample of patients with MSA. However, we also acknowledge some limitations. First, we could not count the specific amount of alcohol consumption of each patient. Second, vascular risk factors were collected from interviews, which could lead to significant recall bias. Third, this was a cross-sectional study. Further prospective, longitudinal follow-up studies are required to confirm our results.

## Conclusion

We found that vascular risk factors were common in patients with MSA. A single vascular risk factor may not show the impact on CI in MSA, however, the cumulative impact of multiple vascular risk factors on CI in MSA should be given proper attention. Patients with MSA may benefit from comprehensive management associated with vascular risk factors.

## Data Availability Statement

The original contributions presented in the study are included in the article/supplementary material, further inquiries can be directed to the corresponding author/s.

## Ethics Statement

The studies involving human participants were reviewed and approved by the Ethics Committee of West China Hospital of Sichuan University. The patients/participants provided their written informed consent to participate in this study.

## Author Contributions

LZ: for the research project: conception, organization, execution; for the statistical analysis: design; for the manuscript: writing of the first draft. YH: for the statistical analysis: review and critique; patients enrollment. BC: for the statistical analysis: review and critique; patients enrollment. Q-QW, RO, JL, KL, TY, YX, and BZ: patients enrollment. HS: for the research project: conception; for the statistical analysis: review and critique; for the manuscript: review and critique. All authors contributed to the article and approved the submitted version.

## Conflict of Interest

The authors declare that the research was conducted in the absence of any commercial or financial relationships that could be construed as a potential conflict of interest.

## Publisher’s Note

All claims expressed in this article are solely those of the authors and do not necessarily represent those of their affiliated organizations, or those of the publisher, the editors and the reviewers. Any product that may be evaluated in this article, or claim that may be made by its manufacturer, is not guaranteed or endorsed by the publisher.

## Abbreviations

MSA: multiple system atrophy
CI: cognitive impairment
PD: Parkinson’s disease
MSA-P: MSA with predominantly parkinsonian features
MSA-C: MSA with predominantly cerebellar ataxia
MRI: magnetic resonance imaging
SCA: spinocerebellar ataxia
UMSARS: unified multiple system atrophy rating scale
OH: orthostatic hypotension
BP: blood pressure
BMI: body-mass index.
